# Supplementary material for: The feasibility and clinical significance of lateral approach thyroidectomy
Source: PLoS One. 2024 Mar 22;19(3):e0300604. doi: 10.1371/journal.pone.0300604 (PMC10959362; doi:10.1371/journal.pone.0300604)
Supplement: S1 Data — (ZIP) [file pone.0300604.s001.zip › ╝╫╫┤╧┘╟╗╛╡╩2╛▌/1111.docx]

1.3观察指标

1.3.1 收集三组患者的年龄、性别、体重指数、肿瘤性质、肿瘤大小等基本情况，肿瘤若为恶性，则需记录清扫的淋巴结数目。

1.3.2 观察并比较三组患者的手术时长、术中出血量、术后引流量、放置引流管时间、住院时间、住院总费用等手术情况。

1.3.3 观察并比较三组患者的声音嘶哑（短暂性）、甲旁减（短暂性）、饮水呛咳、出血、感染、乳糜漏等并发症情况。

1.3.4 术后疼痛评分采用视觉模拟量表（visual analogue scale,VAS），总分10分，分数越低表示疼痛越轻。分别记录术后第1天、第3天患者的疼痛评分。

1.3.5 术后3个月对患者满意度进行随访，满意度评估包括颈前区吞咽功能、皮肤感觉、切口美容情况等，以患者的主观意愿作为评判标准：1分（非常满意），2分（满意），3分（一般），4分（不满意），5分（非常不满意）。

1.4统计学方法

采用IBM SPSS Statistics 25软件进行统计处理，计量资料以（‾x±s）表示，行t检验；计数资料用频数（n）和百分率（%）表示，组间比较采用χ^2^检验。P<0.05表示差异有统计学意义。

**2结果**

3种入路手术组患者均顺利完成手术。

2.1 患者基本情况比较

纳入研究的患者共计52例，其中经锁骨上斜行切口入路甲状腺手术31例，经锁骨下入路腔镜甲状腺手术13例，经腋窝入路腔镜甲状腺手术8例，所有患者均无过胖或过瘦体质。3种入路手术组间患者年龄、体重指数、肿瘤大小差异无统计学意义（P＞0.05）。中央区淋巴结清扫数目差异无统计学意义（P＞0.05），表明3种入路手术组在清扫中央区淋巴结时效果相当。详见表1。

表1 3种入路手术组患者基本情况比较（‾x±s）

| 组别 | 男/女 | 年龄/岁 | 体重指数/（kg/m^2^） | 良性/恶性 | 肿瘤大小 | 淋巴结数目 |
| --- | --- | --- | --- | --- | --- | --- |
| 经锁骨上 |  |  |  |  |  |  |
| 经锁骨下 |  |  |  |  |  |  |
| 经腋窝 |  |  |  |  |  |  |
| F值/t值 |  |  |  |  |  |  |
| P值 |  |  |  |  |  |  |

2.2 患者手术情况比较

手术时间是指从皮肤切开到缝合完毕所用时长，差异具有统计学意义（P＜0.05），说明路径越远，手术耗时越长。3组患者术中均有少量出血，出血量及术后引流量、放置引流管时间、住院时间差异均无统计学意义（P＞0.05）。住院总费用以经腋窝入路组最高，经锁骨下入路组次之，经锁骨上入路组最低。详见表2。

表2 3种入路手术组患者手术情况比较（‾x±s）

| 组别 | 手术时间/min | 出血量/ml | 首日引流量/ml | 总引流量/ml | 放置引流管时间/d | 住院时间/d | 住院总费用/元 |
| --- | --- | --- | --- | --- | --- | --- | --- |
| 经锁骨上 |  |  |  |  |  |  |  |
| 经锁骨下 |  |  |  |  |  |  |  |
| 经腋窝 |  |  |  |  |  |  |  |
| F值 |  |  |  |  |  |  |  |
| P值 |  |  |  |  |  |  |  |

2.3 患者并发症比较

患者术后均未出现伤口出血（包括切口渗血、伤口血肿等）、伤口感染、乳糜漏，3组患者只有经锁骨上入路组出现1例短暂性声音嘶哑，予以营养神经治疗，随访在1月内逐渐恢复，组间差异无统计学意义（P＞0.05）。患者术后可能出现短暂性甲状旁腺功能减退症状，包括不同程度的口唇、手足麻木感，予以补充葡糖糖酸钙后恢复正常，差异无统计学意义（P＞0.05）。喉返神经损伤和甲状旁腺功能减退的发生率很低，可能是细致的解剖所致。其他甲状腺手术相关并发症未发生。详见表3。

表3 3种入路手术组患者并发症比较（‾x±s）

| 组别 | 声音嘶哑（短暂性） | 甲旁减（短暂性） | 饮水呛咳 | 出血 | 感染 | 乳糜漏 | 其他 |
| --- | --- | --- | --- | --- | --- | --- | --- |
| 经锁骨上（n=31) |  |  |  |  |  |  |  |
| 经锁骨下(n=13) |  |  |  |  |  |  |  |
| 经腋窝(n=8) |  |  |  |  |  |  |  |
| χ^2^值 |  |  |  |  |  |  |  |
| P值 |  |  |  |  |  |  |  |

2.4 不同时间段患者疼痛评分比较

3组患者术后第一天切口疼痛都比较明显，高于，差异具有统计学意义（P＜0.05），说明。术后第三天切口疼痛基本缓解，均无剧烈疼痛，VAS评分均有所下降，差异无统计学意义（P＞0.05）。详见表4。

表4 3种入路手术组术后不同时间段切口疼痛评分（‾x±s）

| 组别 | 术后第一天 | 术后第三天 |
| --- | --- | --- |
| 经锁骨上 |  |  |
| 经锁骨下 |  |  |
| 经腋窝 |  |  |
| t值 |  |  |
| P值 |  |  |

2.5 术后3个月患者满意度比较

术后3个月对3组患者进行随访，包括颈前区吞咽功能、皮肤感觉、切口美容情况等。经锁骨上、经锁骨下、经腋窝入路组患者的总体满意度分别为，差异具有统计学意义（P＜0.05），说明切口越隐蔽，患者的满意度越高。详见表5。

表5 3种入路手术组术后3个月满意度比较[n（%）]

| 组别 | 非常满意（1分） | 满意（2分） | 一般（3分） | 不满意（4分） | 非常不满意（5分） | 总体满意度（≤3分） |
| --- | --- | --- | --- | --- | --- | --- |
| 经锁骨上（n=31) |  |  |  |  |  |  |
| 经锁骨下(n=13) |  |  |  |  |  |  |
| 经腋窝(n=8) |  |  |  |  |  |  |
| χ^2^值 |  |  |  |  |  |  |
| P值 |  |  |  |  |  |  |
