# Supplementary material for: The feasibility and clinical significance of lateral approach thyroidectomy
Source: PLoS One. 2024 Mar 22;19(3):e0300604. doi: 10.1371/journal.pone.0300604 (PMC10959362; doi:10.1371/journal.pone.0300604)
Supplement: S1 Data — (ZIP) [file pone.0300604.s001.zip › ╝╫╫┤╧┘╟╗╛╡╩2╛▌/╝╫╫┤╧┘ ╦μ╖├.docx]

甲状腺腔镜随访

| 手术方式 |  |
| --- | --- |
| 姓名 |  |
| 登记号 |  |
| 性别 |  |
| 年龄 |  |
| 声音嘶哑（短暂性） |  |
| 甲旁减（短暂性）口唇、手足麻木感 |  |
| 饮水呛咳 |  |
| 非常满意（1分） |  |
| 满意（2分） |  |
| 一般（3分） |  |
| 不满意（4分） |  |
| 非常不满意（5分） |  |
| 总体满意度（≤3分） |  |
