# Supplementary material for: The feasibility and clinical significance of lateral approach thyroidectomy
Source: PLoS One. 2024 Mar 22;19(3):e0300604. doi: 10.1371/journal.pone.0300604 (PMC10959362; doi:10.1371/journal.pone.0300604)
Supplement: Attachment — Submitted filename: Response to Editor and Reviewers Letter.docx [file pone.0300604.s002.docx]

Dear Editor and Reviewers,

Thank you very much for taking the time to review this manuscript amid your busy schedule. I sincerely appreciate all your comments and suggestions. Below, you will find my itemized responses, and in the resubmitted files, you can locate my revisions/corrections. We have submitted two versions of the manuscript, one with marked changes and the other without markings. Thanks again!

**Responses to Editor**

1. Please ensure that your manuscript meets PLOS ONE's style requirements, including those for file naming.

Author response: Ok, thanks!

2. We note that the grant information you provided in the ‘Funding Information’ and ‘Financial Disclosure’ sections do not match.

When you resubmit, please ensure that you provide the correct grant numbers for the awards you received for your study in the ‘Funding Information’ section.

Author response: Ok, thanks!

3. Please remove your figures from within your manuscript file, leaving only the individual TIFF/EPS image files, uploaded separately. These will be automatically included in the reviewers’ PDF.

Author response: Ok, thanks!

4. We note that Figure 1 in your submission contain copyrighted images. All PLOS content is published under the Creative Commons Attribution License (CC BY 4.0), which means that the manuscript, images, and Supporting Information files will be freely available online, and any third party is permitted to access, download, copy, distribute, and use these materials in any way, even commercially, with proper attribution. For more information, see our copyright guidelines: http://journals.plos.org/plosone/s/licenses-and-copyright.

We require you to either (1) present written permission from the copyright holder to publish these figures specifically under the CC BY 4.0 license, or (2) remove the figures from your submission:

Author response: We change and modify the figure.

**Responses to Reviewer 1**

Reviewer point #1: Authors should report ethical committee approval with code and date of approval.

Author response #1: We added ethics committee approval with code 2023YX134 and an approval date of November 29, 2023. (Line 86-87)

Reviewer point #2: Why authors did not compare the lateral approach with the standard one? Please clarify.

Author response #2: When compared with the standard approach, the lateral approach undoubtedly results in longer procedural time and superior aesthetic outcomes. Therefore, the inclusion of such a comparison might make the study too broad, making it challenging to emphasize key points. The focus of this research is to highlight three pathways that progressively lengthen, step by step, gradually accumulating anatomical experience, with a short learning curve for clinicians. We chose to concentrate on a specific aspect to provide a more in-depth analysis.

Reviewer point #3:  "Of the 52 patients, 8 were male and 44 were female, with an age range of 31-50 years and a mean age of (41.75±9.27) years." This information should be provided in the results section.

Author response #3: This information is provided in the results section. (Line 120-126、Line 183-184)

Reviewer point #4: Where is Weifang People's Hospital located? Please specify.

Author response #4: The location of Weifang People's Hospital is provided in the Correspondence section of the manuscript. (Line 11-13)

Reviewer point #5: Please include the outcomes studied under the subparagraph "outcomes" in the methods section.

Author response #5: The results section contains the full study results, and the methods section contains the observational Indicators related to the results.

Reviewer point #6: In all the tables provided, rows and columns must be inverted, to increase readability.

Author response #6: We have inversion is made for the rows and columns.

Reviewer point #7: In table 3, 4 and 5 there are some characters in chinese. please remove them.

Author response #7: We remove the Chinese characters.

Reviewer point #8: Please report a paragraph with the limitations of the study.

Author response #8: We add the content about the limitations of this study. (Line 388-395)

**Responses to Reviewer 2**

**Introduction:**

Reviewer point #1: Provide more background on the increasing incidence of thyroid cancer and demand for approaches that optimize cosmetic outcomes. Cite doi:10.1007/s00464-010-1341-2.

Author response #1: Please refer to lines 53-55 and Reference 2 for details.

Reviewer point #2: Discuss limitations of conventional thyroidectomy incisions in meeting cosmetic expectations, especially in younger female patients. doi:10.1007/s00464-009-0347-0.

Author response #2: Please refer to lines 358-362 and Reference 23 for details.

Reviewer point #3: Introduce various alternative surgical approaches for thyroid cancer, like transoral, retroauricular etc. and their pros and cons.

Author response #3: Please refer to lines 241-265 in the Discussion section.

Reviewer point #4: Provide more details on the lateral approaches and explain how their incremental dissection could allow sequential accumulation of anatomical experience but increased risk of vocal cord paralysis. cite doi:10.2217/fon-2019-0053

Author response #4: Please refer to lines 353-354 and Reference 22 for details.

Reviewer point #5: Clearly state the rationale and objectives of comparing feasibility and outcomes of the three lateral approaches.

Author response #5: Please refer to lines 60-75 in the manuscript.

**Materials and Methods:**

Reviewer point #1: Expand on inclusion and exclusion criteria for patient selection. Provide the sample size for each surgical approach group.

Author response #1: We have already selected appropriate inclusion and exclusion criteria and provided the sample sizes for each surgical approach group, which are 31, 13, and 8, respectively. (Line 88-90、Line 92-101)

Reviewer point #2: Give more details about surgical equipment used. Specify any specialized instruments.

Author response #2: More details have been provided about the surgical equipment used. (Line 105-114)

Reviewer point #3: Provide more information about general anesthesia protocols and patient positioning for each approach.

Author response #3: Please refer to lines 116-121 in the manuscript.

Reviewer point #4: Explain the step-by-step surgical techniques for each lateral approach, highlighting key anatomical landmarks.

Author response #4: We have made modifications and additions, please refer to lines 122-149 for details.

Reviewer point #5: List all parameters that were observed and recorded during surgery and follow-up.

Author response #5: Please refer to the Results section for details.

**Results:**

Reviewer point #1: Present relevant demographic and clinical characteristics of the patients in a table, with statistical comparisons between groups.

Author response #1: Please refer to Section 2.1 for the comparison of patients' baseline characteristics.

Reviewer point #2: Provide exact p-values and indicators of variance for all numerical outcome comparisons discussed between the groups.

Author response #2: Please refer to the Results section.

Reviewer point #3: Include tables to summarize operative parameters, complications, pain scores, and satisfaction rates, with statistical significance marked.

Author response #3: Please refer to Section 2.2 -2.5.

Reviewer point #4: Comment on any notable patterns and variations between the three groups for the different outcome measures.

Author response #4: There were no significant differences among the three approach groups in terms of patient characteristics, number of central lymph node dissections, intraoperative blood loss, postoperative drainage volume, duration of drainage tube placement, length of hospital stay, postoperative pain, satisfaction, and complications. However, the operation time was longest in the subclavicular approach group, followed by the axillary approach group, and shortest in the supraclavicular approach group. The total hospitalization cost was highest in the axillary approach group, followed by the subclavicular approach group, and lowest in the supraclavicular approach group. Refer to the Results section.

**Discussion:**

Reviewer point #1: Compare your cosmetic and other outcome results with prior studies on lateral and other minimal access thyroidectomy techniques. cite doi:10.1007/s00464-009-0820-9.

Author response #1: Please refer to lines 368-372 and Reference 25 for details.

Reviewer point #2: Discuss possible factors contributing to differences in operation time between the approaches.

Author response #2: Please refer to the Discussion section 3.2 for the analysis of operation time. (Line 317-340)

Reviewer point #3: Interpret the low complication rates in context of meticulous dissection techniques used.

Author response #3: Please refer to lines 248-252 in the Discussion section.

Reviewer point #4: Suggest future research directions, like randomized controlled trials, long-term cosmetic follow-up, application for bilateral thyroidectomy etc.

Author response #4: The study has certain limitations, and future research should focus on large-sample, multicenter studies to achieve broader clinical applicability. (Line 388-395)
