# Supplementary material for: The feasibility and clinical significance of lateral approach thyroidectomy
Source: PLoS One. 2024 Mar 22;19(3):e0300604. doi: 10.1371/journal.pone.0300604 (PMC10959362; doi:10.1371/journal.pone.0300604)
Supplement: Attachment — Submitted filename: Response to Editor and Reviewers Letter (1).docx [file pone.0300604.s003.docx]

Dear Editor and Reviewers,

Thank you very much for taking the time to review this manuscript amid your busy schedule. I sincerely appreciate all your comments and suggestions. Below, you will find my itemized responses, and in the resubmitted files, you can locate my revisions/corrections. We have submitted two versions of the manuscript, one with marked changes and the other without markings. Thanks again!

**Responses to Reviewer 1**

Reviewer point #1: The authors successfully addressed all the comments provided. I believe the manuscript is now ready for acceptance in its present form.

Author response #1: Appreciate your time and valuable input.

**Responses to Reviewer 2**

**Methods:**

Reviewer point #1: Provide more details on patient selection criteria, such as inclusion/exclusion criteria related to tumor stage, disease status, other medical conditions, etc.

Author response #1: The inclusion standards remain unchanged, while exclusion criteria have been added.（Line99-104）

Reviewer point #2: Describe surgical techniques in more granular steps rather than just approach name. Identify key steps like nerve monitoring, hemostasis methods.

Author response #2: Detailed steps for three lateral approach surgical procedures are described.（Line119-152）

Reviewer point #3: Specify equipment/instruments used like endoscopic system, energy devices, monitoring systems, operation table configuration.

Author response #3: The models and manufacturers of the instruments and equipment have been provided. （Line108-117）

Reviewer point #4: Explain outcome measures and how they were assessed - define variables clearly like blood loss (intra-op vs total), complications (grading system), satisfaction scales.

Author response #4: We have explicitly defined variables in the original text. For example, intraoperative blood loss is defined as the volume of bleeding during surgery, and postoperative drainage is defined as the amount of drainage following the operation. All observed complications were transient, and no severe complications occurred; therefore, no grading was performed. The transient nature is explicitly stated in the original text. Satisfaction comparison was assessed through postoperative follow-up scoring on a 5-point scale.（In the results section）

Reviewer point #5: Describe statistical analysis in more detail - what tests were used for which outcomes based on data type/distribution. Specify significance level.

Author response #5: Continuous data were presented as mean ± standard deviation (‾x±s). Analysis of variance (ANOVA) was used for normally distributed data, and non-parametric tests were used for data with non-uniform variances. Categorical data were presented as frequency (n) and percentage (%), and the chi-square test was used for intergroup comparisons. A p-value of less than 0.05 was considered statistically significant.（Line175-181）

**Results:**

Reviewer point #1: Provide summary stats for sample characteristics like mean/median values for age, tumor size rather than just ranges.

Author response #1: In Section 2.1.（Line186-196）

Reviewer point #2: Use tables to present results clearly for easy comparison between groups.

Author response #2: See Results section.

Reviewer point #3: Consider graphical displays like boxplots for non-normally distributed data.

Author response #3: The box plots have been added to the manuscript. （Line208-212、216-217）

Reviewer point #4: Conduct subgroup/stratified analysis where appropriate to identify effect modifiers.

Author response #4: Considering our research objectives, we believe that subgroup/stratified analysis would not be meaningful.

Reviewer point #5: Report both numerical values and percentages for categorical variables like complications.

Author response #5: Refer to Sections 2.3 and 2.5.

Reviewer point #6: Interpret statistically significant vs non-significant findings in the discussion.

Author response #6: Refer to the discussion section 3.1-3.4 for the analysis of the results section.

Reviewer point #7: Highlight key findings and discuss implications for clinical practice and further research.

Author response #7: Refer to lines 391-408.

**Responses to Reviewer 3**

Reviewer point #1: I think authors have addressed the questions raised by the previous reviewers. I have no further comments.

Author response #1: Appreciate your time and valuable input.
